# Pathological grooming: Evidence for a single factor behind trichotillomania, skin picking and nail biting

**DOI:** 10.1371/journal.pone.0183806

**Published:** 2017-09-13

**Authors:** Aniko Maraz, Borbála Hende, Róbert Urbán, Zsolt Demetrovics

**Affiliations:** 1 Institute of Psychology, Eötvös Loránd University, Budapest, Hungary; 2 Doctoral School of Psychology, Eötvös Loránd University, Budapest, Hungary; Technion Israel Institute of Technology, ISRAEL

## Abstract

Although trichotillomania (TTM), skin picking (SP), and nail biting (NB) have been receiving growing scientific attention, the question as to whether these disorders can be regarded as separate entities or they are different manifestations of the same underlying tendency is unclear. Data were collected online in a community survey, yielding a sample of 2705 participants (66% women, mean age: 29.1, SD: 8.6). Hierarchical factor analysis was used to identify a common latent factor and the multiple indicators and multiple causes (MIMIC) modelling was applied to test the predictive effect of borderline personality disorder symptoms, impulsivity, distress and self-esteem on pathological grooming. Pearson correlation coefficients between TTM, SP and NB were between 0.13 and 0.29 (p < 0.01). The model yielded an excellent fit to the data (CFI = 0.992, TLI = 0.991, χ^2^ = 696.65, p < 0.001, df = 222, RMSEA = 0.030, Cfit of RMSEA = 1.000), supporting the existence of a latent factor. The MIMIC model indicated an adequate fit (CFI = 0.993, TLI = 0.992, χ^2^ = 655.8, p < 0.001, df = 307, RMSEA = 0.25, CI: 0.022–0.028, pclose = 1.000). TTM, SP and NB each were loaded significantly on the latent factor, indicating the presence of a general grooming factor. Impulsivity, psychiatric distress and contingent self-esteem had significant predictive effects, whereas borderline personality disorder had a nonsignificant predictive effect on the latent factor. We found evidence that the category of pathological grooming is meaningful and encompasses three symptom manifestations: trichotillomania, skin picking and nail biting. This latent underlying factor is not better explained by indicators of psychopathology, which supports the notion that the urge to self-groom, rather than general psychiatric distress, impulsivity, self-esteem or borderline symptomatology, is what drives individual grooming behaviours.

## Introduction

Many people, even in adulthood, bite their nails, twirl their hair or scratch their skin in moments of stress. These become pathological grooming disorders when they are repetitive and intentional acts of habitual behaviours that result in apparent physical harm and shame due to the inability to control the behaviour [1]. Although trichotillomania, pathological skin picking and nail biting have been receiving growing scientific attention in animal research, especially in rodents, studies in the field of human research are sparse. Furthermore, the question as to whether these disorders can be regarded as separate entities or are different manifestations of the same underlying tendency (pathological grooming) is unclear.

Grooming disorders are relatively common. A recent survey of 1618 people from the United States found that one out of three people met the clinical diagnosis of at least one grooming disorder [2]. This is greater than the prevalence of depression, anxiety or alcohol abuse [3, 4]. Despite this evidence, research generally focuses on grooming in animals, and the studies on human pathological grooming are rare. Furthermore, human studies usually report findings from clinical, treatment-seeking populations; thus, symptom manifestation in non-treatment-seeking individuals is relatively unknown.

The most researched pathological grooming disorder, trichotillomania (TTM, hair-pulling disorder), is characterised by recurrently pulling out one’s hair, which results in hair loss, despite repeated attempts to decrease or stop the behaviour [5]. Those affected pull their hair from anywhere the hair can grow (e.g., scalp, eyebrows, eyelashes and pubic area), and some of these people also ingest the hair pulled. It is estimated to affect at least 3.7 million people in the United States alone, according to conservative estimates [6]. Existing studies indicate that TTM is associated with substantial impairment in daily functioning due to the shame associated with missing hair and bald areas [7]. However, the typical use of small treatment-seeking samples limits complete understanding of the functional impact specific to TTM [8].

According to a recent survey, skin picking (SP, exorciation) was the most frequent impulse control disorder among psychiatric inpatients across Europe, with a lifetime prevalence of 7.3%, which was more than the prevalence of compulsive buying (6.8%), pathological internet use (5.1%) or gambling (2.1%) [9]. SP is characterised by recurrent picking of one’s skin (e.g., to eliminate acne), which results in skin lesions and repeated attempts to decrease or stop the behaviour [5]. Some authors propose that most people engage in SP to some degree. Keuthen et al. [10] found, for example, that 78% of college students (N = 105) engaged in SP at some point in their lives, although the prevalence of clinical level SP is estimated to be “only” 2–4% [10, 11]. Both TTM and pathological SP are classified as obsessive–compulsive and related disorders in the DSM-5.

Nail biting (NB), or onychophagia, is currently a nonofficial diagnostic entity [1]. NB is defined as repetitive biting or chewing of fingernails and occasionally toenails. Apart from possible bacterial infection [12], the behaviour may lead to significant mental distress due to feelings of shame, guilt and malformation [13].

### Grooming disorders: One or three categories?

In the past 10–15 years, researchers have started to recognise the high comorbidity among TTM, SP and NB [1, 14–20]. For example, a review of the literature showed that the prevalence of SP in TTM outpatient samples ranges from 10 to 34% (with an average of 20.8%) and the prevalence of TTM in SP outpatient samples ranges from 5 to 29.2% (average of 15.5%) [17]. Among 38 female patients with TTM, 31% engage in NB and 28% in SP [21]. However, we are unaware of a large-scale nonclinical study that has looked at the comorbidity of TTM, SP and NB.

Furthermore, studies have shown that 3.8–9.5% of SP patients have a family history of TTM, and the prevalence rate of SP in first-degree relatives of individuals with TTM is between 6.6 and 8.3% [17]. Another study showed that concordance rates for monozygotic twins (38.1%) were greater than the concordance rates for dizygotic twins (0%), suggesting that TTM has a significant genetic component with a heritability estimate of 76% [22]. Neuroimaging studies [23, 24] have shown that TTM is associated with abnormalities in the striatum, a brain region where the Sapap3 gene-encoding protein is highly expressed. Furthermore, there is evidence that serotonergic medicines and dopamine-blocking neuroleptics are helpful in treating TTM, NB and in SP [25–28]. This raises the question whether the same underlying disorder (grooming) drives high comorbidity, and, thus, whether TTM, SP and NB can be regarded as three different manifestations of the same pathology.

A possible reason behind the high comorbidity is that grooming disorders are phenomenologically similar to each other. These behaviours are all aimed at removing parts of the body and are triggered by the sight or feel of bodily imperfection, thus, anxiety [1, 14]. Based on clinical samples, several studies have reported increased tension before pulling/picking/biting and gratification or relief during and shortly after the act [29–32]. Furthermore, most people with TTM, SP and NB experience depersonalisation during episodes [33].

Theories that explain the co-occurrence and pathogenesis of grooming disorders are sparse and mainly stem from general theories of psychopathology. This promotes the possibility that the presence of psychiatric distress increases the probability of a comorbid grooming disorder. For example, Flessner et al. [6] reported that as many as 57% of TTM subjects had an additional diagnosis of Axis I disorders, among which major depression (29%) and obsessive–compulsive disorders (11%) were the most common. Similarly high rates of lifetime occurrences of other psychiatric disorders were found in SP [33, 34] and NB [35] subjects as well. Axis II (personality disorders) are also frequently comorbid. According to a study, the most common personality disorders to occur in the presence of SP were obsessive–compulsive personality disorder (48%) and borderline personality disorder (BPD, 26%) [36]. This raises the question: To what extent does psychiatric distress trigger grooming behaviour as a way of reducing anxiety? Finally, there is evidence that low self-esteem [13] and high impulsivity (i.e., the inability to resist urges) [36, 37] also contribute to the development of grooming disorders. However, given that the relationship among personality pathology, psychiatric distress, low self-esteem and impulsivity is remarkably complex [38–42], it is unclear which mechanism is truly underlying a given pathology.

Although grooming disorders have been documented in the scientific literature for over a century, relatively little is known about its characteristics in the normal population. Most studies are based on treatment-seeking populations, which may result in more severe symptom presentation and artificially inflated co-occurrence rates because individuals with multiple symptoms are more likely to seek treatment than those with a single abnormality [43].

The aim of our study was, therefore, to investigate the co-occurrence of pathological grooming behaviours in the community. We wanted to explore whether TTM, NB and pathological SP should be regarded as separate entities or as part of a larger category of grooming disorders. Furthermore, we aimed to ascertain which factors (psychiatric distress, impulsivity, self-esteem or BPD) contribute to the development of pathological grooming. Greater understanding of the relationship not only promotes nosological decisions but also has implications for clinical treatment and may guide research on the etiology.

## Materials and methods

### Participants and procedure

Data were collected in an online survey, which was advertised as “Win three tickets for the Sziget Festival with your habits” and appeared on Hungarian general news and magazine websites. Participants were entered into a price drawing where three incentives (valued at €900) were offered. Participants could reach the survey between January and August 2014. Only individuals older than 18 years could take part. In total, 4177 people participated in the online questionnaire. Data were used if at least 80% of the items were completed, leaving 2875 valid responses. Despite our call, 170 participants were under 18 years old and were consequently excluded. This left 2705 participants’ data for analysis. Out of these, 1026 (36%) intentionally pulled their hair, 2289 (85%) bit their nails and 1198 (44%) intentionally picked their skin at least once during their life. Only those participants who reported to have done the given behaviour at least once were offered to fill out the appropriate grooming questionnaire.

### Instruments

#### Measures of grooming

TTM was assessed by the Massachusetts General Hospital Hairpulling Scale (MGH-HPS). The most widely used TTM instrument, the MGH-HPS is based on the Yale–Brown Obsessive–Compulsive Scale (Y-BOCS) [44]. The MGH-HPS has previously demonstrated strong test–retest reliability (*r* = 0.97) [45]. The questionnaire is self-administered, and participants rate severity, urge to pull, actual pulling, perceived control and associated distress from 0 (no symptom) to 4 (extreme symptom) on a five-point summative response scale. The items were translated and back-translated from English to Hungarian by three independent experts of both languages. The questionnaire provides an estimate of symptom severity in the past seven days. Internal consistency was high in the current sample (*α* = 0.92).

Similar to the MGH-HPS, the Skin Picking Scale-Revised (SPS) was also modelled after the Y-BOCS [10]. The SPS contains eight factors covering impairment and symptom severity. The instrument demonstrated acceptable internal consistency in the current sample (*α* = 0.88).

Considering the lack of clinically adapted nail-biting questionnaires in the literature and in order to comply with the previous questionnaires used in the current study (the MGH-HPS and SPS), we adapted the SPS to measure the severity of NB. The NB scale (NBS) performed acceptable internal consistency (*α* = 0.77) and can be found in the S1 File.

#### Measures of other variables

Impulsivity was measured by the modified Barratt Impulsiveness Scale (BIS, [46]). The 21-item questionnaire has three first-order factors: self-control, impulsive behaviour and impatience. Items are rated 1 to 4. The BIS achieved an acceptable level of internal consistency: α = 0.81.

The Brief Symptom Inventory (BSI) is a 53-item self-report measurement designed to evaluate psychopathology in nine major fields of psychiatry: depression, anxiety, hostility, obsessive–compulsive tendencies, somatisation, phobic anxiety, psychoticism and paranoid ideation [47]. Besides the nine basic dimensions, the Global Severity Index calculates the sum of the scales and four extra items, then divides the sum by the total number of items to which the individual responded. The items are rated on a five-point summative response scale from 0 (not at all) to 4 (very much). The scale was validated in Hungarian [48] and yielded high internal consistency (α = 0.96).

The Zanarini Rating Scale for Borderline Personality Disorder (ZAN-BPD) adapted the BPD criteria from the DSM-IV [49]. Initially developed for clinical use, the instrument was adopted to be used as a self-report measure with nine items. The ZAN-BPD reflects a one-week timeframe, and each of the nine criteria for BPD is rated to be present or absent. The scale had acceptable internal consistency (Cronbach’s α = 0.72).

Contingent self-esteem (CSE) refers to the external sources of a person’s perceived self-worth, such as others’ love and evaluation of competence [50]. Sample items are “I feel worthwhile only when I have performed well” or “I tend to suppress my own needs and emotions to make others feel good”. The 26 items assessing CSE are measured on a scale from 1 to 4, with higher scores indicating increased likelihood to base one’s self-esteem on others’ evaluation. CSE contains two subscales, competence-based and relation-based self-esteem. The scale was translated to Hungarian in the same way as the MGH-HPS. Cronbach’s alpha was 0.93.

### Data analysis

Confirmatory factor analysis with Mplus version 7.3 [51] was used to estimate the degree of fit for each grooming measure (TTM, SP and NB). Four models were tested. Model 1 consisted of correlating first-order factors (TTM, SP and NB defined as latent factors). Model 2 included a hierarchical factor structure representing the general grooming dimension as defined by the three first-order latent factors. Model 3 was a bifactorial model with the three first-order factors and the general latent factor defined by all grooming items. In this model, the correlation between the specific factors and the correlations between the specific factors and the global factor were fixed to zero [52]. Finally, Model 4 was bifactorial with correlating first-order factors.

Due to the severe floor effect in the responses, items were treated as ordinal indicators, and the weighted least squares mean and variance (WLSMV) adjusted estimation method was used [53, 54]. A satisfactory degree of fit requires the comparative fit index (CFI) and the Tucker–Lewis index (TLI) to be higher than or close to 0.95, and the model should be rejected when these indices are less than 0.90 [53, 55]. The next fit index was the root mean square error of approximation (RMSEA). RMSEA below 0.05 indicates an excellent fit, a value around 0.08 indicates an adequate fit and a value above 0.10 indicates a poor fit. Closeness of the model fit using RMSEA (CFit of RMSEA) is a statistical test, which evaluates the statistical deviation of RMSEA from the value 0.05 [53]. A nonsignificant probability value (p > 0.05) indicates an acceptable model fit. Missing data was excluded listwise.

The multiple indicators and multiple causes (MIMIC) modelling technique, a specification of structural equation modelling, was chosen for the present study [56]. We opted for this technique because MIMIC models can estimate the effect of indicators on latent variables when the direct effects of continuous variables on the latent variables are also included. In addition, MIMIC modelling is suitable to validate a construct via reflective modelling [57].

### Ethics

The study protocol was approved by the Institutional Review Board of Eötvös Loránd University and conforms to the Declarations of Helsinki. All participants were informed about the purpose of the study and provided written consent before filling out the questionnaire.

## Results

### Sample description

Out of the total sample (N = 2501), 66% (n = 1650) were women and 34% (n = 851) were men. The mean age was 29.1 years (SD = 8.64). Table 1 depicts most participants as reporting a lifetime occurrence of intentional NB (60%), followed by SP (50%) and TTM (33%). Altogether, 369 (14.7%) participants reported having performed each grooming disorder. Only 441 (17.6%) of the sample reported never having intentionally groomed, and the rest (67.7%) reported two of three behaviours.

**Table 1:** Lifetime occurrence of any intentional grooming behaviour in the sample.

	Trichotillomania	Skin Picking	Nail biting	No grooming
**N**	822 (33%)	1245 (50%)	1497 (60%)	441 (18%)
**% women**	63%	71%	68%	55%
**Mean age**	28.5 (SD:7.95)	27.8 (SD:7.79)	28.4 (SD:8.23)	31.2 (SD:9.9)
**Age of onset (median category)**	13–18 years	13–18 years	0–12 years	-
**Prevalence**^**a**^	Past week: 17% Past month: 5% Past year: 4% Over a year ago: 7%	Past week: 27% Past month: 5% Past year: 4% Over a year ago: 13%	Past week: 19% Past month: 5% Past year: 5% Over a year ago: 31%	-

Note: Question was asked as: “Have you ever pulled your hair / picked your skin / bite your nail?”

^a^Categories were exclusive (e.g., “I have pulled my hair during the past 30 days but not during the past 7 days”).

The Pearson correlation coefficient between TTM and SP was 0.29 (p < 0.001), between TTM and NB was 0.13 (p = 0.005) and between SP and NB was 0.21 (p < 0.001).

### Factor structure of the instruments

Each grooming scale had acceptable fit indices according to the one-factor confirmatory factor analysis regarding the CFI and TLI values. RMSEA was acceptable for SP and NB and well-above the threshold for MGH-HPS (MGH-HPS: χ^2^ = 192.4, df = 13, p < 0.001; CFI = 0.995, TLI = 0.993, RMSEA = 0.130 [0.114–0.146], Cfit < 0.001; SPS: χ^2^ = 226.3, df = 18, p < 0.001; CFI = 0.994, TLI = 0.990, RMSEA = 0.098 [CI: 0.087–0.109], Cfit < 0.001; NBS: χ^2^ = 330.65, df = 18, p < 0.001; CFI = 0.968, TLI = 0.951, RMSEA = 0.086 [CI: 0.078–0.095], Cfit < 0.001).

### Measurement modelling

**Fig 1:**
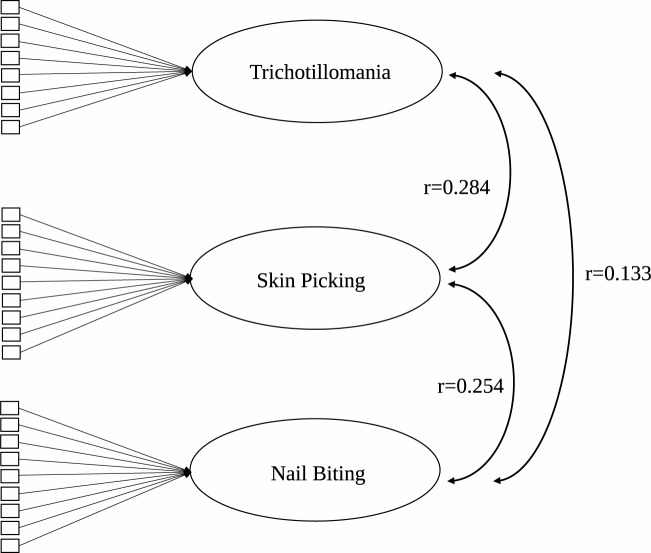
Correlating first-order factors model of grooming. Note: all correlations and loadings are significant on the p < 0.001 level.

**Fig 2:**
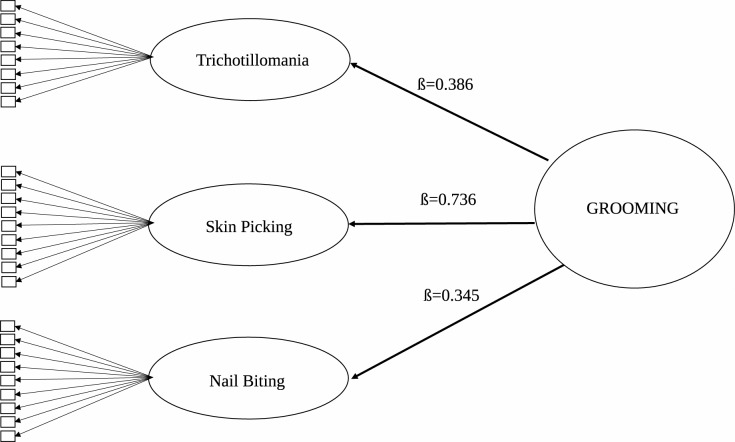
Hierarchical factor model of grooming. Note: all correlations and loadings are significant on the p < 0.001 level.

Both Model 1 (correlating first-order factors, Fig 1) and Model 2 (hierarchical factor analysis, Fig 2) had the same acceptable fit indices (CFI = 0.992, TLI = 0.991, χ^2^ = 696.65, p < 0.001, df = 222, RMSEA = 0.030, Cfit of RMSEA = 1.000) (see Fig 1). In addition, two further bifactorial models were tested. Model 3 (noncorrelating first-order factors plus a general latent grooming factor) had a slightly worse fit than the first two models (CFI = 0.991, TLI = 0.989, χ^2^ = 739.12, p < 0.001, df = 202, RMSEA = 0.033, Cfit of RMSEA = 1.000), but Model 4 (correlating first-order factors + general latent grooming factor) had a better fit than the first three models (CFI = 0.996, TLI = 0.994, χ^2^ = 468.7, p < 0.001, df = 199, RMSEA = 0.030, Cfit of RMSEA = 1.000, WRMR = 1.069).

However, in Model 3 and Model 4, only the minority of grooming items had significant but small factor loadings on the general latent factor (grooming) with loadings between -0.46 and 0.43. Besides being inconsistent in their direction, loadings were small, indicating that grooming items were not predictive of the general latent factor. This result is difficult to interpret and is likely to be a statistical artefactum rather than reflecting true associations. Thus, for reasons of interpretability, we decided to choose the next best-fitting model, which was Model 2. This model still had excellent fit indices and yielded interpretable findings.

In Model 2, all items loaded significantly on their respective factor. Loadings were between 0.72 (associated distress) and 0.97 (frequency of urges) for MGH-HPS, between 0.56 (“Have you been avoiding doing anything, going any place or being with anyone because of your skin picking? If yes, then how much do you avoid?”) and 0.93 (“How much time do you spend picking your skin per day?”) for SPS, and between 0.57 (“Do you chew and swallow pieces of your nail after biting?”) and 0.78 (“How frequently do you regret biting your nail?”) for NBS.

### MIMIC model

**Fig 3:**
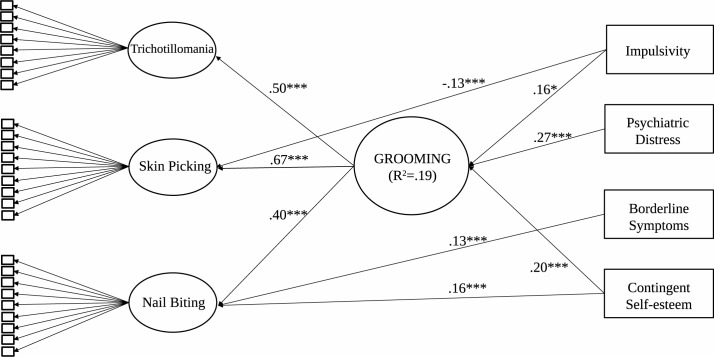
The MIMIC model of pathological grooming and indicators of psychopathology. Note: *p < 0.05, ***p < 0.01, n.s. = nonsignificant. Only significant (p < 0.05) path coefficients are included in the model for reasons of clarity.

In order to test grooming disorders and the latent factor and indicators of psychopathology, a MIMIC analysis was carried out. The MIMIC model yielded an adequate fit to the data based on Model 2 (CFI = 0.993, TLI = 0.992, χ^2^ = 655.8, p < 0.001, df = 307, RMSEA = 0.25, CI: 0.022–0.028, pclose = 1.000). As shown in Fig 3, TTM, SP and NB each loaded significantly on the latent factor, indicating the presence of a general grooming factor. Impulsivity, psychiatric distress and contingent self-esteem had a significant predictive effect, whereas borderline personality disorder had a nonsignificant predictive effect, on the latent factor. However, impulsivity predicted SP, whereas borderline symptoms and self-esteem predicted NB. TTM was not predicted by any of the current indicators of psychopathology.

## Discussion

In the current study, we found evidence that a category of pathological grooming is meaningful and encompasses three symptom manifestations: trichotillomania, skin picking and nail biting. The latent underlying factor is not better explained by indicators of psychopathology, which supports the notion that the urge to self-groom, rather than general psychiatric distress, is what drives individual grooming behaviours. Furthermore, pathological grooming is related to impulsivity, psychiatric distress and low self-esteem, in this order or strength.

This study is in line with previous findings which support the notion that OCD-related conditions appear to be strongly related to each other, as evidenced by high rates of comorbidity, heredity, shared phenomenological features and obsessive thoughts [58, 59]. Overlap (common variance) between TTM, SP and NB behaviours is due to grooming tendency because the association between each grooming behaviour and the latent factor is stronger than between indicators of pathology and the latent factor. This indicates that the common underlying construct is grooming rather than the general psychopathology or method effect [60].

Self-directed behaviour can be used as a behavioural indicator of stress and anxiety in animals, especially in primates [61]. Furthermore, empirical evidence shows that grooming results in soothing [62, 63]. For example, rats increase self-grooming behaviour as a result of stress caused by novelty [64]. The current study supports the generalisability of this notion in humans. It is possible that increased psychiatric distress (and to a lesser extent, regret due to the lack of self-control) is manifested via self-directed repetitive behaviour, which is either TTM, SP or NB. Thus, in the absence of control over the outside world, grooming behaviour provides temporary relief from stress. Nevertheless, it is unclear to what extent this effect is specific to grooming. There is evidence that psychosocial stress is predictive of future depressive symptoms and symptom severity in OCD (and Tourette syndrome) [65], and the current study did not screen for the presence of these disorders. These and other disorders might also be linked through deficits in the Sapap3 protein [66]. Therefore, future studies should confirm the model in other obsessive–compulsive spectrum disorders and control for the presence of Tourette syndrome.

The negative association between self-esteem and grooming is not without precedent in animal research. For example, subordinate females in macaque monkeys showed higher rates of self-scratching than dominant ones, especially when giving and receiving aggression [65]. This is in line with our findings in humans because people who perceive themselves low in the social hierarchy (low self-esteem) groom themselves more than those in a higher position.

The finding that increased impulsivity predicts grooming behaviour is in line with previous results. Neurocognitive studies in adults with TTM have demonstrated deficits in inhibitory control and response flexibility [66] and motor inhibition [67, 68]. The novelty in the current findings is that impulsivity predicts pathological grooming independent from other related factors, such as psychiatric distress. Perhaps this mechanism explains the beneficial effect of N-acetyl cysteine (NAC) medication (a modulator of the glutamatergic system involved in behavioural control) in the treatment of SP [69] and grooming [70].

Contrary to our expectations, BPD seems unrelated to pathological grooming. Our previous hypothesis was based on the finding that nonoptimal mother–infant bonding, a major risk factor of BPD, is associated with decreased levels of oxytocin later in life [71]. Given that the level of oxytocin plays a crucial role in self-mutilating behaviour [72, 73], the assumption that BPD and grooming are related seemed plausible. In addition, there is already evidence that oxytocin administration reduces repetitive behaviour in autism spectrum disorders [74]. The lack of association between BPD symptoms and pathological grooming in the current study is perhaps due to the fact that the effect of the borderline personality model is mediated through impulsivity and ineffective coping mechanisms (distress), which are both central features of BPD. Therefore, future studies should explore the role of personality pathology (especially BPD) and pathological grooming in more detail.

This study is not without its limitations. It is possible, for example, that only those individuals who are aware of and ready to report their condition participated in the study, which might have led to self-selection bias. Another limitation is the ignorance of the heterogeneity of the condition. Future studies should control for subtypes of each condition. Finally, given the cross-sectional nature of the study, it is difficult to ascertain causality.

In conclusion, we found evidence for the existence of pathological grooming based on phenomenological exploration. Thus, in the future, it might be more meaningful to assess pathological grooming tendency rather than individual conditions (TTM, SP or NB) which are “only” manifestations of the same underlying condition: bodily focused repetitive self-harming. Given that this is the first study to report findings from a large community sample, future studies should confirm the findings.

## Supporting information

S1 File The nail biting questionnaire. (PDF)
