# Supplementary material for: Pathological grooming: Evidence for a single factor behind trichotillomania, skin picking and nail biting
Source: PLoS One. 2017 Sep 13;12(9):e0183806. doi: 10.1371/journal.pone.0183806 (PMC5597179; doi:10.1371/journal.pone.0183806)
Supplement: S1 File — (PDF) [file pone.0183806.s001.pdf]

S1 File. The nail biting questionnaire.

Please note that all questions are based on the Massachusetts General Hospital (MGH) hairpulling scale.
See: Keuthen, N., O'Sullivan, R., Ricciardi, J., Shera, D., Savage, C., Borgmann, A., . . . Baer, L. (1995). The Massachusetts General Hospital (MGH) hairpulling scale: 1. development and factor analyses. *Psychotherapy and Psychosomatics*, *64*(3-4), 141-145.

Instructions: *For each question, pick the one statement in that group which best describes your behaviors and/or feelings over the past week. If you have been having ups and downs, try to estimate an average for the past week. Be sure to read all the statements in each group before making your choice.*

For the next three questions, rate only the urges to bite your nails.

1. [**Frequency of urges]** On an average day, how often did you feel the urge to bite your nails?

0 This week I felt no urges to bite my nails.
1 This week I felt an **occasional** urge to bite my nails.
2 This week I felt an urge to bite my nails **often**.
3 This week I felt an urge to bite my nails **very often.**
4 This week I felt **near constant** urges to bite my nails.

2. [**Intensity of urges]** On an average day, how intense or "strong" were the urges to bite your nails?

0 This week I did not feel any urges to bite my nails.
1 This week I felt **mild** urges to bite my nails.
2 This week I felt **moderate** urges to bite my nails.
3 This week I felt **severe** urges to bite my nails.
4 This week I felt **extreme** urges to bite my nails.

3. [**Ability to control the urges]** On an average day, how much control do you have over the urges to bite your nails?

0 This week I could **always** control the urges, or I did not feel any urges to bite my nails.
1 This week I was able to distract myself from the urges to bite my nails **most of the time.**
2 This week I was able to distract myself from the urges to bite my nails **some of the time.**
3 This week I was able to distract myself from the urges to bite my nails **rarely.**
4 This week I was **never** able to distract myself from the urges to bite my nails.

For the next three questions, rate only the actual nail biting.

4. [**Frequency of nail biting]** On an average day, how often did you actually bite your nails?

0 This week I did not bite my nails.
1 This week I bit my nails **occasionally.**
2 This week I bit my nails **often.**
3 This week I bit my nails **very often.**
4 This week I bit my nails so often it felt like I was **always** doing it.

5. [**Attempts to resist nail biting]** On an average day, how often did you make an attempt to stop yourself from actually biting your nails?
    - 0 This week I felt no urges to bite my nails.
    - 1 This week I tried to resist the urge to bite my nails **almost all of the time.**
    - 2 This week I tried to resist the urge to bite my nails **some of the time.**
    - 3 This week I tried to resist the urge to bite my nails **rarely**.
    - 4 This week I **never** tried to resist the urge to bite my nails.

6. [**Control over nail biting]** On an average day, how often were you successful at actually stopping yourself from biting your nails?
    - 0 This week I did not bite my nails.
    - 1 This week I was able to resist bite my nails **almost all of the time.**
    - 2 This week I was able to resist bite my nails **most of the time.**
    - 3 This week I was able to resist bite my nails **some of the time.**
    - 4 This week I was **rarely** able to resist bite my nails.

For the last question, rate the consequences of your nail biting.

7. [**Associated distress]** Nail biting can make some people feel moody, "on edge," or sad. During the past week, how uncomfortable did your nail biting make you feel?
    - 0 This week I did not feel uncomfortable about my nail biting.
    - 1 This week I felt **vaguely uncomfortable** about my nail biting.
    - 2 This week I felt **noticeably uncomfortable** about my nail biting.
    - 3 This week I felt **significantly uncomfortable** about my nail biting.
    - 4 This week I felt **intensely uncomfortable** about my nail biting.
